# Opportunities for big data in conservation and sustainability

**DOI:** 10.1038/s41467-020-15870-0

**Published:** 2020-04-24

**Authors:** Rebecca K. Runting, Stuart Phinn, Zunyi Xie, Oscar Venter, James E. M. Watson

**Affiliations:** 1 School of Geography, The University of Melbourne, Parkville, VIC Australia; 2 School of Earth and Environmental Sciences, The University of Queensland, Brisbane, QLD Australia; 3 Natural Resource and Environmental Studies Institute, University of Northern British Columbia, Prince George, BC Canada; 4 Wildlife Conservation Society, Global Conservation Program, New York, NY USA

**Keywords:** Environmental sciences, Policy

## Abstract

Big data reveals new, stark pictures of the state of our environments. It also reveals ‘bright spots’ amongst the broad pattern of decline and—crucially—the key conditions for these cases. Big data analyses could benefit the planet if tightly coupled with ongoing sustainability efforts.

This big data revolution, which encompasses techniques to capture, process, analyse and visualize large datasets in a rapid timeframe, has led to an explosion in data variety over the last five decades (Fig. 1a). Significant advances in data growth in the bio-geophysical sciences have allowed scientists to discover, analyse and understand environmental changes at micro to global scales, and separate out what is human-driven. As a consequence, the state and trends of the environment is increasingly becoming a focus of big data applications (Fig. 1b). Here, we discuss the trends emerging from these environmental analyses (including the derived data products) and propose a way forward to harness these technologies to mitigate global environmental declines.

**Fig. 1:**
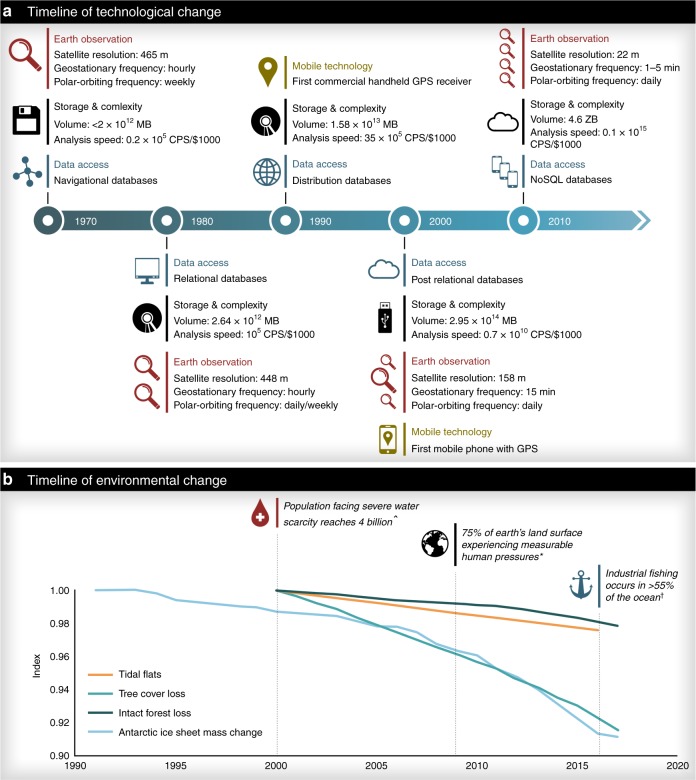
Timeline of selected technological and environmental changes. (**a**) The volume of data produced has grown exponentially and is expected to soon reach 40 Zettabytes (40 trillion Gigabytes). Such data generation is only possible due to the concurrent growth in data storage and computing speed, which has moved from the floppy disk (~1 calculations per second per $1000) to cloud-based storage (>10^15^ calculations) in last 30 years^1^. Despite this exponential growth in technological capacity, and increasing environmental applications, our planet is still facing serious environmental declines (**b**). All environmental declines shown are sourced from prior studies (as detailed below) and are indexed relative to their state in the first year plotted (i.e., dividing by the first value in each time series), with the exception of Antarctic ice sheet mass change^2^, which was indexed against expected (BAU) loss by 2100 (81 cm sea-level rise equivalent^3^). Tidal flats represent the overall decline across the globe for time period, and does not show annual fluctuations^4^. Intact Forest Landscapes and tree cover loss does not take into account gains^5,6^. Note the index on the *y*-axis is only shown for the range 0.9–1. ^Ɨ^Data from Global fishing watch^7^. *Based on the global human footprint^8^. ^For at least 1 month a year over the period 1996–2005^9^.

## Environmental changes revealed by big data

Almost invariably, the advances in big data analyses confirm planetary declines and, in most cases, reveal that declines are worse than previously indicated (Fig. 1b). For example, a landmark collaboration between NASA and the European Space Agency integrated the Antarctic ice sheet’s changing volume, flow and gravitational attraction to model its surface mass balance, which revealed Antarctica lost 2720 ± 1390 Mg of ice between 1992 and 2017 (equating to a sea-level rise of 7.6 ± 3.9 mm)^2^. Yet only a few years prior, the IPCC stated that ‘we have medium confidence in model projections of a future Antarctic SMB [surface mass balance] increase, implying a negative contribution to GMSL [global mean sea level] rise’ (p.1155^10^). Similarly, efforts to create a Red List of Ecosystems—an emerging methodology using multiple lines of evidence to assess the threat status of ecosystems^11^—has already revealed some alarming results. For instance, in the Americas and the Caribbean, 85% of the forest area and 80% of forest types are potentially threatened^12^. While these methodical developments are impressive and urgently needed, they reveal a stark picture for the environment.

However, analyses of big data have also revealed ‘bright spots’ amongst the broad pattern of decline and—crucially—identify the key drivers, including deliberate policy interventions. For instance, while Hansen et al.^5^ revealed dramatic declines in forest extent across the globe, forest loss in Brazil was decreasing by 1318 km^2^ y^−1^ through the 12 year period to 2012, primarily due to a progressive legal framework covering forests during the study period (although the change in government in 2019 has since reversed this trend). Similarly, recent analyses of satellite data by Chen et al.^13^ showed that direct human land-management has led to greening over large expanses in China and India. Much of the gains in China were from forest (rather than agriculture), which was driven by ambitious national policies for afforestation and forest conservation underpinned by payments for these ecosystem services^14^. Biophysical drivers can also produce positive trends. For instance, an analysis of derived big data products revealed that increased precipitation in the Tibetan Plateau over the last four decades has resulted in vegetation greening^15^. While this greening is good for carbon sequestration, it is important to note that it may ultimately disrupt the existing ecosystem and species. Overall, the mean human pressure in the world’s 24 most developed countries decreased from 1993-2009, potentially due to rural-to-urban migration and restoration programs^8^. Despite these regional improvements, they are not yet sufficient to reverse the global trend towards environmental decline.

## Coupling big data and the global sustainability agenda

Analyses of big data are clearly essential for highlighting declines in Earth’s environment and its capacity to support humans. Yet, these impressive advances will not benefit the planet and its people unless we can act to achieve sustainability goals, so it is essential that big data coalesce with ongoing efforts to achieve sustainability. Unfortunately, this is not yet the case for many national and international policy processes. Despite international agreement on the Aichi Biodiversity Targets (which form the strategic plan for the Convention for Biological Diversity), many nations are not taking advantage of the availability of big data and derived products to achieve these goals. Numerous spatial data layers necessary for implementing the Aichi Targets already exist at the national or global scale, yet 80% of the 5th round of National Reports on these targets contained no actionable maps^16^. In most cases, a map of environmental phenomena requires additional analysis and combination with other datasets before it can be used to directly inform decisions, which may require additional resources (e.g., technical, financial) that are challenging for some less-developed countries to acquire.

It is essential that barriers to analysing big data and accessing derived products are removed. This could take the form of accessible outputs, such as the Global Fishing Watch, Global Forest Watch and global inter-tidal websites (www.globalfishingwatch.org, www.globalforestwatch.org, www.intertidal.app), or user-friendly analysis tools, such as REMAP – a free application that utilises the storage and analysis capacity of Google Earth Engine to map land cover change (www.remap-app.org). While there is a growing trend towards open data, some authorities still maintain barriers to access including fees, substantial delays, or incomplete data releases. For example, in the Australian state of Queensland, which has one of the most dramatic recent land clearing legacies, governmental reporting on vegetation clearance is often delayed by months despite the fact it could be monitored monthly. Ensuring the timely availability of big data products will require a sincere commitment to a shared vision for open data, before more procedural issues such as data management standards can be addressed. A number of international organisations are addressing this, with the WMO (World Meteorological Office, https://public.wmo.int/), possibly one of the longest running and best examples of what is possible from real global collaborations across many countries, while the GEO (Group on Earth Observations, www.earthobservations.org) links 108 nation’s earth observation capabilities to address sustainable development goals. Further, as international accords for conservation and sustainability are operationalised by countries, it is vital to engage national- and international-level decision-makers in government and industry in the generation of global datasets to ensure they are able to be used by all to make effective decisions.

The private sector is increasingly making influential environmental decisions and some large companies are committing to sustainability in their supply chains. Examples include ‘zero-deforestation’ and sustainably sourced palm oil pledges from Nestlé and McDonalds. Tracking the full supply chain for large corporations requires the use of big data analytics, particularly to balance the multiple objectives corporations seek from their supply chains (such as reducing carbon emissions and increasing profitability)^17^. The use of geospatial, earth observation and many other data will be essential for transparency and monitoring compliance by certification bodies, environmental NGOs and the corporations themselves. Ongoing research, particularly integrating qualitative data, is likely to enable an even closer coupling of big data analytics and sustainable supply chain management. Ultimately, the timely use of big data will be critical to placing commodity production and trade on a sustainable pathway.

Big data now must be harnessed for ecological forecasting to improve decision-making, in both the public and private sectors. Monitoring environmental change in near real-time can be beneficial if there is capacity for action at a similar temporal scale, which is often not the case. However, useful applications are emerging. For instance, Chen et al.^18^ investigated links between sea surface temperatures and interannual changes in fire activity in South America and forecasted the regional severity of the fire season with a 3–5 month lead time. Across the Middle East, Levin and colleagues^19^ integrated temporal data on night lights, wildfire, news databases and Flickr photos to identify World Heritage Sites affected by conflict in near real-time. In the marine realm, an automated vessel tracking and monitoring system (which uses a constellation of satellites and terrestrial receivers) can be used to inform models, which predict illegal fishing activity in real-time^20^. Identifying patterns of suspicious behaviour has allowed governments to conduct targeted investigations of particular vessels that may be undertaking illegal activity in their waters. Such ‘early warning systems’, should be given a platform in relevant management agencies—and closely linked with management action—to harness vast potential of these methods to improve outcomes for nature and people.

To ensure these close links with environmental decision-making, the acquisition and analysis of big data must be solution focused and address sustainability challenges while engaging with decision-makers and those affected by such decisions. From documenting our planet’s greenness to detecting where resources are being illegally harvested, big data analyses can now place detailed evidence of rapid environmental change in the hands of entities capable of management action. Ultimately, there must be a tight coupling of big data analyses and the sustainability agenda to ensure we do not run out of time and space to save our environment—and ourselves.
